# Contradictory Effects of NLRP3 Inflammasome Regulatory Mechanisms in Colitis

**DOI:** 10.3390/ijms21218145

**Published:** 2020-10-30

**Authors:** Kohei Wagatsuma, Hiroshi Nakase

**Affiliations:** Department of Gastroenterology and Hepatology, Sapporo Medical University School of Medicine, Minami 1-jo Nishi 16-chome, Chuo-ku, Sapporo, Hokkaido 060-8543, Japan; waga_a05m@yahoo.co.jp

**Keywords:** inflammasome, NLRP3, inflammatory bowel disease, colitis, autophagy, microbiota

## Abstract

The inflammasome is an intracellular molecular complex, which is mainly involved in innate immunity. Inflammasomes are formed in response to danger signals, associated with infection and injury, and mainly regulate the secretion of interleukin-1β and interleukin-18. Inflammasome dysregulation is known to be associated with various diseases and conditions, and its regulatory mechanisms have become of great interest in recent years. In the colon, inflammasomes have been reported to be associated with autophagy and the microbiota, and their dysregulation contributes to colitis and. However, the detailed role of inflammasomes in inflammatory bowel disease is still under debate because the mechanisms that regulate the inflammasome are complex and the inflammasome components and cytokines show seemingly contradictory multiple effects. Herein, we comprehensively review the literature on inflammasome functioning in the colon and describe the complex interactions of the NOD-like receptor family pyrin domain-containing 3 (NLRP3) inflammasome components with inflammatory cytokines, autophagy, and the microbiota in experimental colitis models and patients with inflammatory bowel disease.

## 1. Introduction

The innate immune system is activated when a pattern recognition receptor (PRR) sensor recognizes a danger signal. Danger signals that are common to pathogens are referred to as pathogen-associated molecular patterns (PAMPs). In recent years, it has become clear that some PRRs also recognize danger-associated molecular patterns (DAMPs), which are secreted by stressed and injured cells and cause an aseptic inflammatory reaction. Therefore, the pattern recognition mechanism is involved not only in acute inflammation, which is associated with pathogen infections, but also in inflammation associated with various chronic inflammatory diseases and bacteria inside and outside the body. The inflammasome is an intracellular molecular complex, which is mainly involved in innate immunity and is formed in response to danger signals associated with infection and injury. Inflammasome dysregulation is known to be associated with various diseases and conditions, and its regulatory mechanism has become of great interest in recent years. The concept of the inflammasome was proposed in 2002 based on the analysis of cryopyrin-associated periodic syndrome (CAPS), a hereditary autoinflammatory disorder [1]. In 2006, it was reported that inflammation in gout attacks was mediated by inflammasome activation by urate crystals, which became a focus of attention as a new molecular mechanism of aseptic inflammation [2].

PRRs aggregate with apoptosis-associated speck-like protein containing a caspase recruitment domain (CARD) (ASC) and caspase-1 to form the inflammasome, which induces caspase-1 activation. The activated caspase-1 induces cell death, called pyroptosis, and cleaves precursors of inflammatory cytokines, such as interleukin (IL)-1β and IL-18, to induce the extracellular release of their mature forms [3,4,5,6]. Cytokine secretion is known to be associated with cases of pyroptosis due to caspase-1 activation or extracellular release of organelles. Caspase-1 cleaves gasdermin D to form cell membrane pores, causing pyroptosis, which is accompanied by the outflow of cytoplasmic components and induces an inflammatory response by releasing mature inflammatory cytokines [7]. Thus, inflammasomes play a role in defense against infection by inducing both immune activation by inflammatory cytokines and the elimination of infected cells via pyroptosis.

Several types of inflammasomes have been reported so far, among which the NOD-like receptor (NLR) family pyrin domain (PYD)-containing 3 (NLRP3) inflammasome is activated not only by PAMPs but also by DAMPs and can cause aseptic inflammation in various diseases, such as gout and arteriosclerosis. In the intestine, inflammasomes are not only important mediators of the host defense but also important regulators of intestinal homeostasis, which regulate the defense function of the intestinal epithelium and the immune response to the gut microbiota [8,9,10]. Dysregulation of the inflammasome is known to be associated with various diseases. In the intestine, inflammasomes are known to be associated with autophagy and the microbiota, and dysregulation of inflammasomes contributes to the development of inflammatory bowel disease (IBD). Among all kinds inflammasome, the NLRP3 inflammasome is the most characterized one. The NLRP3 inflammasome, has been analyzed extensively in its contribution to colitis and is considered to be important in the development of therapeutic strategies. However, the detailed role of inflammasomes in IBD is still under debate because the mechanism that regulates the inflammasome is complex and the inflammasome components and cytokines show seemingly contradictory multiple effects. In addition, the contribution of NLRP3 inflammasome to intestinal inflammation is complicated, because recent experimental data suggest that the manipulation of microbiota in NLRP3 knockout (KO) mice cause contradictory data. Herein, we comprehensively review the literature on inflammasome functioning in the colon and describe the complex interactions of the NLRP3 inflammasome components with inflammatory cytokines, autophagy, and the microbiota in experimental colitis models and patients with IBD. Moreover, we summarized the results regarding the control methods performed in several studies associated with NLRP3 inflammasome for aiming to avoid the influence of the microbiota.

## 2. Structure of the Inflammasome

The basic structure of the inflammasome consists of a PRR as a sensor molecule, ASC as an adapter molecule, and caspase-1 [11]. PRRs include Toll-like receptors (TLRs), NLRs, the retinoic acid-inducible gene-I-like receptor (RLR), and the pyrin and HIN domain (PYHIN) receptor family which includes the absent in melanoma 2 (AIM2) -like receptor (ALR) and C-type lectins. In the NLR subfamily, NLRP1, NLRP3, NLR family CARD-containing 4 (NLRC4), NLRP6, and NLRP12 are commonly known [12,13].

**Figure 1:**
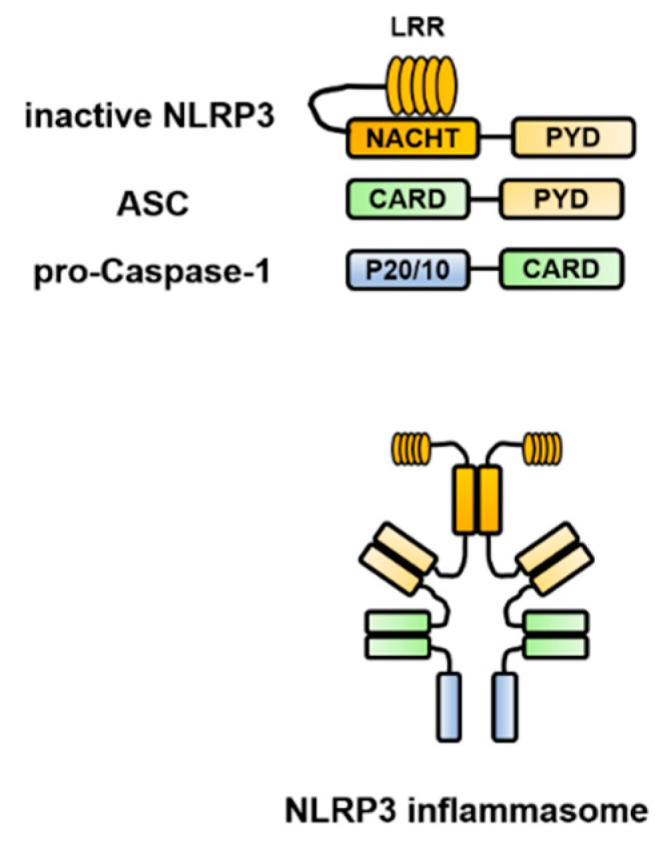
Structure of the NOD-like receptor family pyrin domain-containing 3 (NLRP3) inflammasome. NLRP3 self-polymerizes via the NACHT [NAIP (neuronal apoptosis inhibitory protein), CIITA (MHC class II transcription activator), HET-E (incompatibility locus protein from *Podospora* anserina) and TP1 (telomerase-associated protein)] domain and binds to apoptosis-associated speck-like protein containing a caspase recruitment domain (ASC) via the pyrin domain (PYD). Furthermore, ASC binds to caspase-1 via the caspase recruitment domain (CARD) to form a complex, which brings the caspase-1 precursors into close proximity to each other, leading to their self-activation and processing of pro-interleukin (IL)-1β and pro-IL-18 into their mature forms. ASC, apoptosis-associated speck-like protein containing a caspase recruitment domain; CARD, caspase recruitment domain; IL, interleukin; LRR, leucine-rich repeat; NLRP3, NOD-like receptor family pyrin domain-containing 3; NACHT, (neuronal apoptosis inhibitory protein, MHC class II transcription activator, incompatibility locus protein from *Podospora* anserina, and telomerase-associated protein); PYD, pyrin domain.

Each of the constituent molecules of the inflammasome has a PYD and CARD, which are domain structures responsible for the characteristic intermolecular interactions and interact in a homophilic binding mode. For example, the structure of the NLRP3 inflammasome is presented in Figure 1. In the NLRP3 protein, there are three core domains [1,2]. The NACHT [NAIP (neuronal apoptosis inhibitory protein), CIITA (MHC class II transcription activator), HET-E (incompatibility locus protein from *Podospora* anserina), and TP1 (telomerase-associated protein)] domain promotes self-oligomerization and ATPase activity. NACHT is required for the activation of inactive procaspase-1 into active caspase-1 and subsequent homo- or heterooligomerization, leading to autocleavage and secretion of IL-1β and IL-18. The N-terminus contains a PYD, which binds to caspase-1 via an adapter protein (ASC) to form a complex. The C-terminus contains a leucine-rich repeat (LRR), which plays an essential role in the responsiveness to danger signals. Inflammasome assembly is inhibited by the LRR domain, whose activity is neutralized by the activation signal from PAMPs or DAMPs. ASC includes two transduction domains, a PYD, which can connect to the upstream NLRP3, and a CARD, which can connect to the downstream caspase-1.

## 3. Control Mechanism of the NLRP3 Inflammasome Activation

### 3.1. Two-Signal Control of the NLRP3 Inflammasome Activation

**Figure 2:**
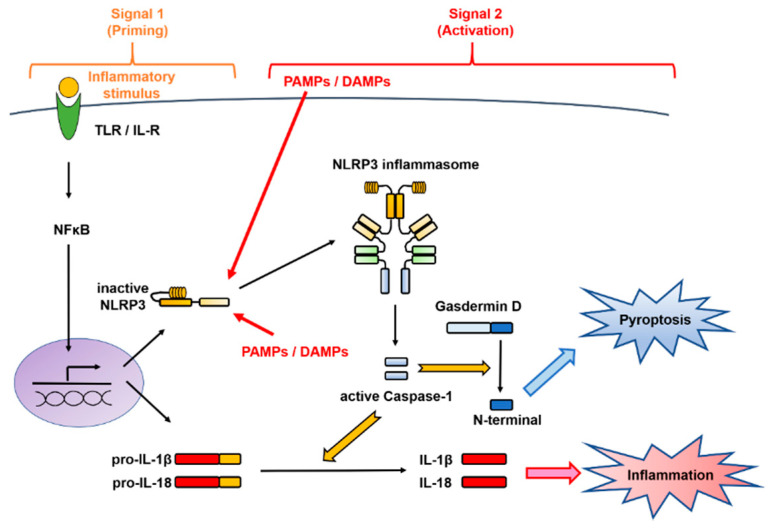
Two-signal control of the NOD-like receptor family pyrin domain-containing 3 (NLRP3) inflammasome activation. Activation of the NLRP3 inflammasome implicates a first signal, called priming, which requires an inflammatory stimulus involved in transcriptional induction, and a second signal, called triggering, which requires a danger signal involved in posttranslational regulation. In the first stage is priming signal for inflammasome activation. mRNA expression of the interleukin (IL-1β)- and NLRP3-encoding genes from Toll-like receptor (TLR) or the IL-1 receptor (IL-1R) through nuclear factor-kappa B (NF-κB) and NLRP3 protein expression by deubiquitination are induced. In the second stage, microbial or danger signals can directly activate inflammasome assembly. DAMPs, danger-associated molecular patterns; IL, interleukin; IL-1R, interleukin-1 receptor; NF-κB, nuclear factor-kappa B; NLRP3, NOD-like receptor family pyrin domain-containing 3; PAMPs, pathogen-associated molecular patterns; TLR, Toll-like receptor.

The activation of the NLRP3 inflammasome implicates a first signal, called priming, which requires an inflammatory stimulus involved in transcriptional induction, and a second signal, called triggering, which requires a danger signal involved in posttranslational regulation [8,11,14] (Figure 2). These mechanisms are distinctive for the IL-1 family and are different from those involved in the production of other cytokines.

### 3.2. NLRP3 Inflammasome Regulators

**Figure 3:**
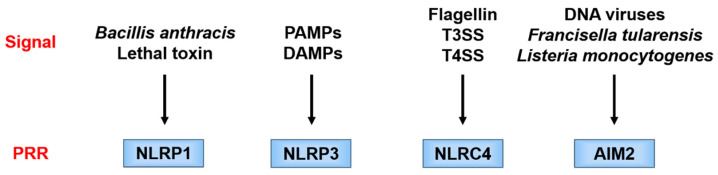
Danger signals recognized by different pattern recognition receptor (PRRs). Each PRR recognizes a different danger signal. NOD-like receptor family pyrin domain-containing 1 (NLRP1) is activated by certain bacterial toxins. NOD-like receptor family caspase recruitment domain-containing 4 (NLRC4) works in combination with neuronal apoptosis inhibitory protein (NAIP), and the ligand itself binds to NAIP. NAIP recognizes the bacterial protein flagellin and the constituent proteins of the type III secretion system (T3SS) of Gram-negative bacteria and forms a complex with NLRC4. Absent in melanoma 2 (AIM2) directly recognizes double-stranded DNA and is considered to be activated during infection with double-stranded DNA viruses, such as cytomegalovirus, and cell-invasive bacteria, such as *Francisella tularensis* and *Listeria monocytogenes*. AIM2, absent in melanoma 2; DAMPs, danger-associated molecular patterns; T3SS/T4SS, type III/IV secretion system; PAMPs, pathogen-associated molecular patterns; PRR, pattern recognition receptor; NLRC4, NOD-like receptor family caspase recruitment domain-containing 4; NLRP1/NLRP3, NOD-like receptor family pyrin domain-containing 1/3; NAIP, neuronal apoptosis inhibitory protein.

Each PRR recognizes a different danger signal [15,16] (Figure 3). Since pathogen-derived PAMPs mainly activate these PRRs, the latter are considered to function as a defense against infection. On the other hand, NLRP3 is activated not only by PAMPs but also by endogenous or exogenous DAMPs and, therefore, is considered of importance in aseptic inflammation. Since there are various stimulators of NLRP3, it is considered that NLRP3 is activated by recognizing a common signal induced by danger signals.

**Figure 4:**
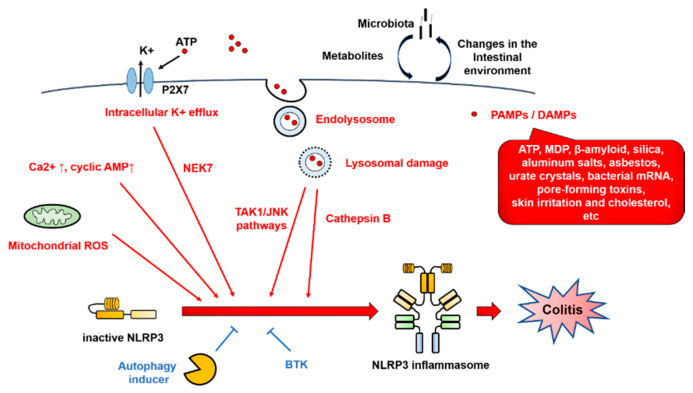
NOD-like receptor family pyrin domain-containing 3 (NLRP3) inflammasome regulators. NLRP3 inflammasome activation involves various types of pathogen-associated molecular patterns (PAMPs) or danger-associated molecular patterns (DAMPs), such as ATP, muramyl dipeptide (MDP), β-amyloid, silica, asbestos, urate crystals, bacterial mRNA, pore-forming toxins, skin irritants, and cholesterol. In addition, the major mechanistic pathways and stimuli that trigger NLRP3 inflammasome activation have been reported to include intracellular K^+^ efflux, mitochondrial reactive oxygen species (ROS), and lysosomal damage. Red arrows indicate activation of NLRP3 inflammasome. Blue T-shaped lines indicate inhibition of NLRP3 inflammasome activation. BTK, Bruton’s tyrosine kinase; DAMPs, danger-associated molecular patterns; JNK, c-Jun N-terminal kinase; MDP, muramyl dipeptide; NEK7, NIMA-related kinase 7; NLRP3, NOD-like receptor family pyrin domain-containing 3; PAMPs, pathogen-associated molecular patterns; ROS, reactive oxygen species; TAK1, TGF-β-activated kinase 1.

NLRP3 inflammasome activation involves various types of PAMPs or DAMPs [4,17]. In addition, the major mechanistic pathways and stimuli that trigger NLRP3 inflammasome activation have been reported to include intracellular K^+^ efflux, mitochondrial reactive oxygen species (ROS), and lysosomal damage [14] (Figure 4).

In the intracellular K^+^ efflux pathway, extracellular ATP, which is released from dead cells, causes K^+^ efflux via the P2 × 7 receptor, and selective K^+^ efflux is also caused by bacterial toxins, resulting in a decrease in the intracellular K^+^ concentration and activation of NLRP3. By contrast, NLRP3 activation is blocked by inhibiting K^+^ efflux from cells [18]. Meanwhile, the opening of large mitochondrial pores is not required for NLRP3 activation [19].

Lysosomal damage is triggered by endogenous uric acid crystals and cholesterol crystals or by exogenous particles, such as silica and asbestos. NLRP3 is activated by the intracellular release of cathepsin B, which is a lysosomal enzyme. The TGF-β-activated kinase 1 (TAK1)/c-Jun N-terminal kinase (JNK) pathway, which is a mitogen-activated protein kinase signaling pathway, is activated via lysosomal disruption, and this kind of activation is required for full activation of the NLRP3 inflammasome [20]. Silica crystals and aluminum salts activate NLRP3 inflammasomes via destabilization of phagosomes. Acidification of phagosomes or inhibition of cathepsin B activity impairs NLRP3 activation [21].

ROS derived from organelles positively regulate NLRP3 inflammasome activity. Mitochondria are a major intracellular source of ROS, and thus, mitochondrial function plays an important role in modulating inflammation. Mitochondrial ROS are produced when inhibition of the electron transport chain or removal of abnormal mitochondria via autophagy (mitophagy) is impaired [22]. Mitochondrial ROS are required for non-transcriptional priming of NLRP3, and NLRP3 deubiquitination is a prerequisite for activation [23]. On the other hand, it has been reported that ROS inhibitors block priming of the NLRP3 inflammasome but not its activation [24].

In addition, NIMA-related kinase 7 (NEK7), protein kinase R, and guanylate-binding protein 5 (GBP5) link these common pathways with NLRP3 [11,25]. NEK7 is an essential protein that acts downstream of the K^+^ efflux pathway to mediate NLRP3 inflammasome assembly and activation [26]. NEK7 is involved in the regulation of the cell cycle, mitotic spindle formation, and cytokinesis. Activation of the NLRP3 inflammasome by NEK7 is restricted to the interphase of the cell cycle [27].

Other regulatory mechanisms have been reported to involve NLRP3 phosphorylation [28] and ubiquitination [29], as well as the calcium-sensing receptor (CaSR). The CaSR modulates NLRP3 inflammasome activation via changes in intracellular Ca^2+^ and cyclic AMP concentrations [30]. Activation of proteasome- and lysosome-dependent mechanisms by the CaSR promotes the degradation of key modulators of NLRP inflammasome activation [31]. Recently, Bruton’s tyrosine kinase (BTK) has been reported to play a role in physiological inhibition of NLRP3 inflammasome activation, which explains why patients with X-linked agammaglobulinemia tend to develop Crohn’s disease (CD) [32]. The platelet-activating factor receptor regulates colitis-induced lung inflammation via the NLRP3 inflammasome [33]. Kynurenic acid/GPR35 axis limits NLRP3 inflammasome activation and exacerbates colitis in socially stressed mice [34]. These findings suggest that NLRP3 inflammasome activation involves a wide range of regulatory mechanisms.

### 3.3. Non-Canonical Inflammasomes

In recent years, new protein complexes, called non-canonical inflammasomes, have ben discovered. Caspase-11 plays a central role in mice, while caspase-4 and caspase-5 play a central role in humans. Caspase-11 is critical for caspase-1 activation and IL-1β production in macrophages infected with *Escherichia coli* and *Citrobacter rodentium* [35,36]. Lipopolysaccharide (LPS) or outer membrane vesicle derived from Gram-negative bacteria enter host cells via several different routes, leading to the direct interaction with caspase-11 to form LPS-caspase-11 complexes. This promotes inflammasome-independent pyroptosis and proteolytic activation of caspase-1 and activation of caspase-1 to cleave pro-IL-1β and pro-IL-18 into secreted bioactive cytokines [37].

## 4. Diseases Associated with Inflammasome Dysfunction

An inflammasome with an adequate range of activities functions as a self-defense mechanism and contributes to the prevention of external pathogen invasion. However, inflammasome dysfunction can trigger or exacerbate various types of inflammatory and immune-related diseases [38,39,40,41]. The NLRP3 inflammasome is activated not only by pathogens but also by air pollutants, such as asbestos and silica particles, which cause respiratory diseases [42]. In addition, metabolites that are accumulated in the body as a result of overnutrition and β-amyloid that is accumulated in the brain unduly activate the NLRP3 inflammasome. Inflammasome dysfunction has been found to play a central role in the development and progression of various diseases, such as gouty arthritis [2], type 2 diabetes [43], atherosclerosis [44], ischemic heart disease [45], rheumatoid arthritis [46], Alzheimer’s disease [47], and stroke [48]. However, the responsible DAMPs for each disease are often unknown.

**Figure 5:**
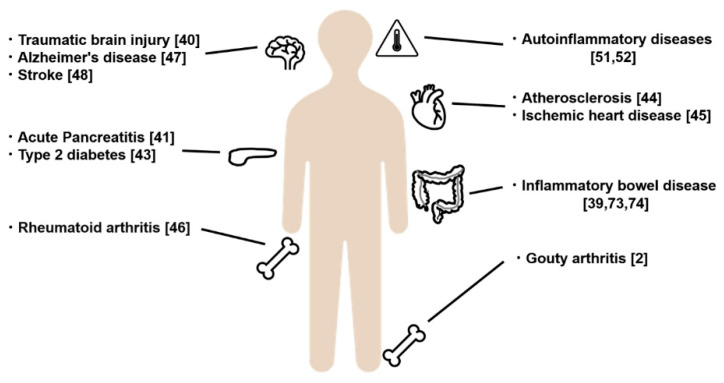
Diseases associated with inflammasome dysfunction. NOD-like receptor family pyrin domain-containing 3 (NLRP3) inflammasome dysfunction can trigger or exacerbate various types of inflammatory and immune-related diseases in different organs. NLRP3, NOD-like receptor family pyrin domain-containing 3.

Targeting the inhibition of the NLRP3 inflammasome contributes to the relief of diseases [45,48]. Inhibition of IL-1β may also affect lung cancer development and mortality, independent of the suppression of cardiovascular events [49] (Figure 5). However, inhibition of IL-1β may also lead to the exacerbation of infection and delay tissue repair.

### 4.1. Autoinflammatory Diseases

Autoinflammatory diseases cause recurrent systemic inflammation. In addition to the typical symptoms of periodic fever, symptoms such as skin rash, arthralgia, and serositis, which are similar to those of infectious and collagen diseases, are observed. However, no pathogenic microorganisms have been identified, and no autoantibodies or antigen-specific T cells have been detected [50]. These symptoms are due to a functional abnormality in cells of the innate immune system, accompanied by the activation of antigen-independent inflammatory cells. At the center of the pathophysiology of an autoinflammatory disease is the overproduction of IL-1β and IL-18, which is associated with abnormal activation of the inflammasome. Autoinflammatory diseases are broadly classified into inflammasome-related and unrelated [51,52].

Activation of the NLRP3 inflammasome is highly involved in the etiology and pathology of familial Mediterranean fever (FMF) and CAPS. FMF is a hereditary autoinflammatory disease characterized by periodic fever attacks of relatively short duration, and abdominal and chest pain associated with serositis. The *MEFV* gene in the short arm of chromosome 16 (16p13.3) has been identified to be responsible for FMF [53]. Pyrin, which is encoded by the *MEFV* gene, negatively regulates the NLRP3 inflammasome. Excessive inflammation is induced via increased production of inflammatory cytokines such as IL-1β and IL-18 [54,55,56]. On the other hand, it has been suggested that the activation of a pyrin inflammasome via a gain-of-function pyrin mutation also contributes to the pathophysiology of FMF [57]. Thus, the roles of *MEFV* mutations and pyrin dysfunction are still controversial.

FMF is mainly characterized by serositis and is rarely accompanied by intestinal mucosal lesions. However, in recent years, the number of cases with intestinal mucosal lesions has been increasing, and FMF-related enterocolitis should be distinguished from ulcerative colitis (UC) or CD [58,59,60,61,62,63].

### 4.2. IBD

IBD is usually classified into two subtypes, UC and CD. UC affects the mucosa and submucosa of the colon. CD may affect any region of the gastrointestinal tract, and the lesions consist of granulomatous inflammatory lesions with ulcers and fibrosis [64]. The exact etiology of IBD remains unclear, however, it is considered to be caused by environmental factors, genetic factors, microbial factors, and immune system dysfunction in the intestinal mucosa [65,66]. The mechanisms of chronic, recurrent inflammation may lead to the onset and progression of IBD [67].

Recently, it has been increasingly reported that the NLRP3 inflammasome is involved in the pathogenesis and progression of IBD [39,68,69]; however, the role of the NLRP3 inflammasome in IBD is still controversial.

## 5. Factors Associated with the NLRP3 Inflammasome in IBD

### 5.1. Cytokines and Pyroptosis

It is speculated that IL-1β is mainly involved in the early stage of intestinal inflammation. However, favorable effects of IL-1β and IL-18 on the repair and recovery of the ulcerative epithelium have also been reported. On the other hand, excessive production of IL-18 may enhance chronic inflammation and promote tumor formation and growth [70]. Furthermore, the cleavage of gasdermin D by caspases and the resulting pyroptosis have also been the focus of attention. Pyroptosis is an important innate effector mechanism that normally protects the host from intracellular pathogenic infections; however, its dysfunction has been suggested to cause intestinal inflammation. In IBD, it is necessary to comprehensively investigate these effectors.

#### 5.1.1. IL-1β

A recent genome-wide association study meta-analysis has shown that single-nucleotide polymorphisms (SNPs) in *IL18R1*, *IL1R1*, *IL1RL1*, *IL1RL2*, and *IL1R2* are associated with susceptibility to IBD [71]. IL-1β is mainly produced by natural immune cells [72] and may trigger a variety of processes in both immune and non-immune cells, including proliferation, differentiation, and apoptosis [73,74]. IL-1β can induce local mucosal immune responses by stimulating T-cell proliferation and can direct neutrophils to the site of injury or infection [75,76]. Furthermore, IL-1β stimulates the production of IL-6 as a growth factor for B-cell proliferation and initiates the release of other proinflammatory cytokines, such as tumor necrosis factor (TNF)-α and IL-23. According to the antigenic environment, IL-1β polarizes the adaptive immune response toward a T helper (Th) 2 or Th17 profile [5,77].

Previous reports have shown overproduction of IL-1β in patients with IBD and in mouse models, suggesting the progression of IL-1β-induced mucosal inflammation [77,78,79]. Normal colon macrophages only stimulate the production of the precursor form of IL-1β upon LPS stimulation, whereas IBD colon macrophages can release mature IL-1β [79]. It has been implicated that patients with IBD may have a mucosal imbalance of intestinal IL-1 and IL-1 receptor (IL-1RA), and insufficient production of the endogenous IL-1RA may cause the pathogenesis of chronic intestinal inflammation [78]. Dextran sulfate sodium (DSS)-induced severe colitis in mice has been suggested to be due to the activation of the NLRP3 inflammasome and release of mature IL-1β [80].

On the other hand, recent findings have suggested that increased IL-1β production plays a protective role against colitis. It was reported that IL-1β could prevent intestinal *C. rodentium* and *Clostridium difficile* infections by stimulating bacterial phagocytosis by mononuclear phagocytes [81]. Furthermore, transplantation of mesenchymal stem cells pretreated with IL-1β was shown to reduce DSS-induced colitis [82]. NLRP3 KO mice were demonstrated to be more sensitive to oxazolone treatment, showing reduced production of mature IL-1β and IL-18; this phenotype was rescued by exogenous IL-1β or IL-18 [83].

#### 5.1.2. IL-18

It has been reported that polymorphisms in *IL18* are associated with increased susceptibility to CD, especially among Asians and Africans [84,85]. IL-18 is a multifunctional cytokine, which is constitutively present in all cells. Recent studies have shown that epithelial IL-18 secretion is independent of NLRP3 but dependent on caspase-1 [86]. However, as NLRP3 plays one of the most important roles in caspase-1 activation, it is possible that NLRP3 also contributes to IL-18 production in the intestine. IL-18 functionally induces interferon (IFN)-γ and promotes the Th1 response [87]. The activity of IL-18 is balanced by the IL-18-binding protein (IL-18BP). Free IL-18 is significantly elevated in children with CD, suggesting an inadequate compensatory increase in its natural inhibitor, IL-18BPa [88]. On the other hand, IL-18 is able to induce the secretion of IFN-γ by Th1 and natural killer (NK) cells and regulate the growth and repair response in the intestinal tract when the epithelium is damaged.

It has been reported that IL-18 is upregulated in patients with IBD, and high levels of IL-18 were detected in lamina propria mononuclear cells and intestinal epithelial cells (IECs) [89,90]. However, these studies were unable to determine whether the increased IL-18 levels were the cause or consequence of IBD pathophysiology. In a mouse model of colitis, neutralization of IL-18 was shown to reduce IFN-γ production and improve intestinal inflammation [91,92]. Deletion of *Il18* or its receptor *Il18r1* in IECs protected mice from mucosal damage. By contrast, the deletion of *Il18bp* caused severe colitis, which was associated with the loss of mature goblet cells [93].

There have been numerous reports suggesting that IL-18 could suppress colitis [83,94], which indicates that IL-18 may be involved in the repair of the intestinal epithelial layer by maintaining an appropriate level of epithelial cell proliferation during acute experimental colitis.

#### 5.1.3. Pyroptosis

Pyroptosis is a special form of programmed inflammatory cell rupture, playing an important role in antibacterial innate immune defense. Pyroptosis is promoted by the activation of procaspase-1 by some pathogens [95]. Activated caspase-1 processes gasdermin D, and its N-terminus forms a pore in the cell membrane, causing pyroptosis. Recent findings have suggested that cleavage of gasdermin D by mouse caspase-11 or human caspase-4 is also essential for pyroptosis [96]. Pyroptosis removes the replicative niche of intracellular pathogens, making pathogens more susceptible to phagocytosis and killing by secondary phagocytes. However, abnormal systemic activation of pyroptosis in vivo may contribute to sepsis [97].

In a mouse model of spontaneous colitis deficient in both of the two UC susceptibility genes, *TLR2* and *MDR1A*, myeloid CD11b^+^ cells overreacted to nonpathogenic *E. coli* or LPS, and activation of the inflammasome caused myeloid CD11b^+^ cells to undergo pyroptosis. In a study of patients with UC who carried *TLR2* and *MDR1* mutations, increased caspase-1 activation and cell death were observed in the inflamed colonic layer [98].

In a recent study, Bulek et al. [99] have reported that epithelium-derived gasdermin D mediates nonlytic IL-1β release during experimental colitis and that gasdermin D deficiency substantially attenuates colitis severity; in this study, heterozygous control littermates (Gasdemin D^+/−^) were used. In contrast, Ma et al. [100] reported that gasdermin D in macrophages restrains colitis by controlling cyclic GMP-AMP synthase-mediated inflammation and that gasdermin D deficiency in macrophages leads to exacerbated experimental colitis; in this study, littermate WT controls were used.

### 5.2. Autophagy

Autophagy is a basic cell survival process that plays a broad homeostatic role by removing toxic waste products, including factors of excessive inflammation [101]. Recently, several studies have demonstrated the importance of autophagy in the protection against chronic inflammatory diseases [102]. Autophagy dysfunction is deeply related to the pathology of IBD, especially CD. In addition, autophagy has been found to be an essential process in controlling excessive inflammasome activation. A specific allele of the autophagy-related gene *ATG16L1* has been suggested to be associated with impaired autophagy and increased IL-1β production upon NLRP3 inflammasome activation [103]. It has also been suggested that NLRP3 inflammasome activation is triggered by mitochondrial damage and excess ROS production [4].

Activation of autophagy by inflammatory signals limits IL-1β production by targeting ubiquitinated inflammasomes [104]. In addition, autophagy regulates inflammation by interacting with innate immune signaling pathways, eliminating endogenous inflammasome agonists, and affecting the secretion of immune mediators [101]. Autophagy deficiency interferes with organelle clearance and induces abnormal activation of inflammasomes, leading to severe tissue damage. Autophagy deficiency also leads to an abnormal increase in IL-1β levels [104] and is especially profound in DSS-induced acute colitis [105]. These results suggest that the NLRP3 inflammasome activity is negatively regulated by autophagy. Furthermore, defects in autophagy disrupt the morphology of and secretion by secretory granules in both Paneth and goblet cells, which results in changes in the inflammatory microenvironment [106].

The function of ATG16L1 has also been investigated in bone marrow cells [105]. ATG16L1-deficient macrophages were shown to produce large amounts of IL-1β and IL-18 [105]. Impairment of autophagy using an *ATG5*-specific small interfering RNA or an autophagy inhibitor (3-methyladenine) induced LPS/DSS-induced NLRP3 inflammasome activation in peritoneal macrophages [107].

Furthermore, autophagy mediates the degradation of mitochondrial and inflammasome components, including NLRP3, to suppress unnecessary and abnormal activation of inflammasomes. Consequently, blockade of mitophagy and autophagy leads to the accumulation of mitochondrial ROS and activation of the NLRP3 inflammasome [108].

In addition, metabolites such as uric acid crystals, cholesterol crystals, and free fatty acids, when taken up by macrophages, damage phagolysosomes and further damage mitochondria. When damaged mitochondria generate ROS, the NLRP3 inflammasome is activated [108]. The microbiota and its metabolites can also directly affect autophagy [109].

On the other hand, autophagy is also involved in the secretion of cytokines, such as IL-1β and IL-18 [110,111], and can cause the degradation of the inflammasome components NLRP3, AIM2, and pro-IL-1β [112].

Thus, autophagy is involved in both positive and negative innate immune responses by controlling the NLRP3 inflammasome.

### 5.3. Microbiota

**Figure 6:**
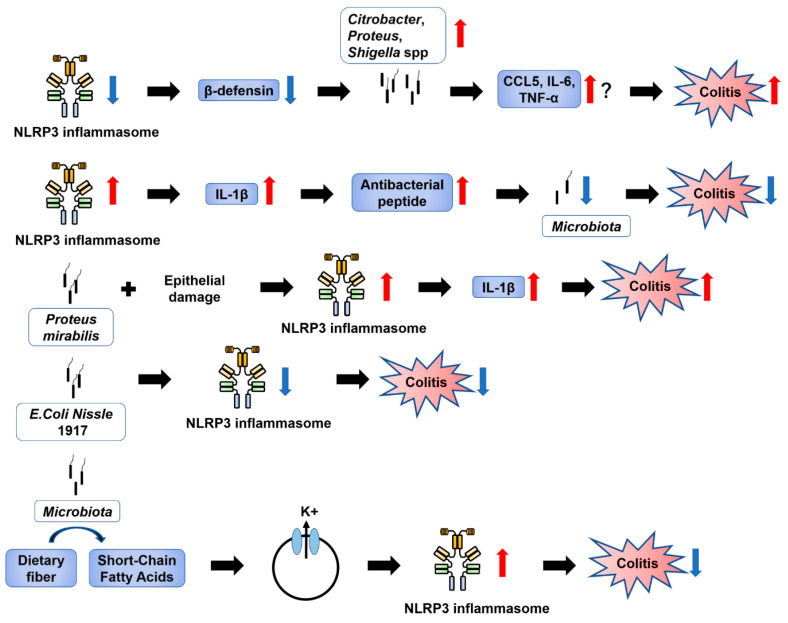
Relationship between NOD-like receptor family pyrin domain-containing 3 (NLRP3) inflammasome and microbiota. NLRP3 inflammasome intestinal inflammation via the microbiota. The microbiota controls intestinal inflammation via the NLRP3 inflammasome. In addition, short-chain fatty acids produced by the microbiota induce NLRP3 inflammasome activation. IL, Interleukin; NLRP3, NOD-like receptor family pyrin domain-containing 3; TNF, Tumor necrosis factor.

Microbiota signaling pathways are essential for the normal development and homeostasis of the gastrointestinal mucosa. The microbiota and microbial metabolites have been reported to be involved in intestinal inflammation and carcinogenesis [113]. Microorganisms may cause excessive or persistent inflammation in genetically susceptible individuals [114]. Therefore, many studies have focused on the importance of the microbiota as a target for therapeutic interventions. Several studies have reported the detrimental effects of NLRP3 inflammasome dysfunction on microbial homeostasis (Figure 6).

#### 5.3.1. Involvement of NLRP3 in Intestinal Inflammation via Effects on the Microbiota

Inhibition of NLRP3 affects the microbiota and makes the intestine more susceptible to colitis. In NLRP3 KO mice, with increased susceptibility to colitis, the development of potentially pathogenic bacteria, such as *Citrobacter*, *Proteus*, and *Shigella* spp., was observed, and Changes in β-defensin levels were most likely responsible for changes in microbiota composition [115]. However, molecular pathways via which the microbiota of NLRP3 inflammasome-deficient mice promotes intestinal inflammation have not been confirmed. It has been speculated that the cause is the ability to upregulate the production of pro-inflammatory chemokines and cytokines such as CCL5, IL-6, and TNF-α [116,117].

In addition, Yao et al. [86] found that an overactive NLRP3 inflammasome, which led to local overproduction of IL-1β, could confer strong resistance to experimental colitis and CAC. Increased IL-1β levels enhanced the production of local antibacterial peptides to promote microbiota remodeling. The anti-inflammatory capacity may have also depended on the abundance of T regulatory (Treg) cell-promoting bacteria, such as *Clostridium* XIVa and *Lactobacillus murinus.*

#### 5.3.2. Regulation of Intestinal Inflammation by Microbiota via NLRP3

There are many reports suggesting that the microbiota activates NLRP3 and exacerbates colitis. *Proteus mirabilis* can normally be found in the intestinal lumen but does not trigger an inflammatory response. In the presence of epithelial damage, e.g., caused by DSS treatment, *P. mirabilis* may pass through the damaged epithelial layer and reach recruited inflammatory macrophages. *P. mirabilis* induces potent IL-1β secretion via NLRP3 inflammasome activation in Ly6C^high^intestinal Ly6C^high^ monocytes [118]. One of the pathological factors of CD is the presence of adhesion-infiltrating *E. coli*, which induces IL-1β secretion via an NLRP3-dependent mechanism [119]. Additionally, fecal bacteria in patients with CD were shown to upregulate the expression of NOD2, NLRP3, TLR2, and TLR4 [120].

Meanwhile, it has been reported that some members of the microbiota can protect against colitis by suppressing NLRP3 activation. The probiotic *E. coli* Nissle 1917 has a significantly lower ability to activate inflammasomes than does the commensal *E. coli* K12 [121], while showing the ability to ameliorate human UC [122] and mouse DSS-induced colitis [123].

#### 5.3.3. Short-Chain Fatty Acids (SCFAs)

The microbiota supports the host by breaking down dietary fiber into short-chain fatty acids (SCFAs). A dietary fiber-deprived intestinal microbiota degrades the colonic mucus barrier, increases the host’s susceptibility to pathogens, and promotes epithelial access and deadly colitis caused by the mucosal pathogen *C. rodentium* [124]. Butyrate, one of the major SCFAs, induces differentiation of mouse colon Treg cells [125]. The presence of dietary fiber and SCFAs protects against DSS-induced colitis via NLRP3 activation in colonic epithelial cells. SCFAs that bind to GPR43 in colonic epithelial cells stimulate K^+^ efflux and hyperpolarization, leading to NLRP3 inflammasome activation [110].

## 6. Inflammasome Dysfunction in the Colon

### 6.1. Colitis

#### 6.1.1. Patients with IBD

There are many reports on the association between *NLRP3* polymorphisms and IBD [126]. Genome-wide association studies have found that loss-of-function *NLRP3* polymorphisms are associated with CD development. The presence of SNPs in the regulatory region of the *NLRP3* gene has been associated with susceptibility to CD [69]. One of the *NLRP3* SNPs, the risk allele of rs6672995 was associated with a decrease in LPS-induced IL-1β production in peripheral blood mononuclear cells (PBMCs) [69]. In a recent case-control study, the *NLRP3* SNP rs10754558 was found to be significantly associated with UC [127]. It has been reported that the “GG” genotype of rs10754558 may be associated with the presence of UC, as this disease is common among patients with the “GG” genotype. On the other hand, the “CG” genotype may be associated with a protective effect against UC. Functional analysis has shown that the risk alleles of rs10754558 increased the enhancer activity of *NLRP3* mRNA stability since the G allele increases the *NLRP3* expression compared to the C allele [128].

CARD8 is one of the negative regulators of the NLRP3 inflammasome. CARD8 binds to NLRP3 during inflammasome activation, prevents its binding to its downstream adaptor ASC and the assembly of the NLRP3 inflammasome [129]. The V44I mutation in the T60 isoform of *CARD8*, which is a loss-of-function mutation of *CARD8*, is associated with enhanced IL-1 production and increased NLRP3 inflammasome activation. Patients with this mutation increased the severity of CD and were not effectively treated with anti-TNF-α antibodies but responded to IL-1β inhibitors [130]. In a study of Korean patients with CD and UC, only the *CARD8* intron 1 rs1972619 SNP was associated with CD. The rs2043211 stop allele was associated with elevated serum IL-1β levels only in female patients with UC [131]. In this study, no relationship was observed between these *CARD8* polymorphisms and the clinical phenotype of UC or CD. Bagnall et al. [132] reported that CD patients carrying the stop allele of rs2043211 express an immunoreactive isoform of *CARD8* and a somewhat reduced level of its mRNA. However, many studies have reported the association between the *CARD8* gene polymorphism rs2043211 and the susceptibility to CD, but the results have remained quite contradictory. Zhang et al. [133] reported that no statistically significant association was found between the *CARD8* polymorphism and CD risk in the overall population. On the other hand, they reported that stenotic or fistulizing CD, the mutant-type rs2043211 polymorphism may generate a potentially protective effect.

On the other hand, a relatively large UK cohort study failed to demonstrate a significant genetic association between *NLRP3* SNPs and susceptibility to CD [134]. A Korean study tested four SNPs with unknown biological functions in the *NLRP3* gene and found no statistically significant association with IBD [131]. In a Swedish cohort, there was no significant association between NLRP3 inflammasome-associated *EIF2AK2* gene polymorphisms and the onset or clinical outcome of IBD [135]. In a recent study from China, the *NLRP3* gene was associated with UC but not with CD [136]. In a study from Greece, patients with CD showed activation of the NLRP3 inflammasome by PBMC stimulation [137]; however, no significant difference was detected between the UC and control groups. Among patients with UC, NLRP3 activation was associated with long-standing disease.

#### 6.1.2. Experimental Models

- Caspase-1

In early reports, caspase-1 is often involved in the exacerbation of colitis. Caspase-1 KO mice showed a reduction of acute and chronic DSS-induced colitis [138]. Treatment with caspase-1 inhibitors alleviated DSS-induced colitis and showed that IL-18-mediated cytokine production was an important factor in DSS-induced colitis [139]. IL-1β protein levels were increased in IL-10 KO mice, but a caspase-1 inhibitor significantly improved spontaneous colitis in these mice [140].

By contrast, another study showed that caspase-1 KO mice had increased susceptibility to DSS-induced colitis, and the severity of colitis was associated with a decrease in IL-18 production [141]. Caspase-1 KO mice showed defects in mucosal tissue repair, and the administration of exogenous IL-18 completely reversed the severity of colitis.

- NLRP3

Some studies reported that NLRP3 inflammasome activation was detrimental to the progression of colitis in a DSS-induced mouse model [142,143]. Lesions were significantly less severe in caspase-1- or NLRP3-deficient mice than in wild-type (WT) mice and were associated with decreased levels of IL-1β and IL-18 [138,144]. Recently, oral administration of titanium dioxide nanoparticles, which are widely used in food additives or pharmaceutical formulations, was reported to exacerbate acute colitis via a mechanism involving the NLRP3 inflammasome [145]. These results suggest that excessive activation of NLRP3 exacerbates colitis.

By contrast, there are many reports showing that NLRP3 has a function of protecting against colitis. Oral administration of DSS to NLRP3-deficient mice was reported to have resulted in a loss of epithelial integrity, systemic dispersal of the microbiota, and more severe colitis. This process was the result of a decrease in IL-18 [94]. NLRP3 KO mice were found to be susceptible to DSS- and 2,4,6-trinitrobenezene sulfonic acid-induced experimental colitis and had reduced levels of IL-1β, IL-10, and TGF-β. Furthermore, changes in β-defensin expression, decreased antibacterial secretion, and changes in the microbiota were observed in the colon [115].

Moreover, the activation of NLRP3 prevents colitis. Patients with CAPS who carry *NLRP3* mutations do not experience self-inflammation in the intestine, suggesting that NLRP3 has a protective effect on the intestine. In addition, in contrast to a gene-deficient model, the NLRP3 R258W mouse model displays enhanced NLRP3 inflammasome signaling, increased secretion of IL-1β, but not IL-18, and an enhanced Th17 cell-dominant immune response [146].

#### 6.1.3. Therapy

Many new therapies that target the inhibition of the NLRP3 inflammasome have been reported for the treatment of IBD. Administration of MCC950 to spontaneous colitis mice (Winnie mouse) protected against colitis [147]. In a DSS-induced mouse model, alpinetin [143] could prevent colitis. Soy isoflavones were shown to ameliorate [148], while 1,25(OH)_2_D_3_ [149] and flavonoid VI-16 [150] were shown to protect against experimental colitis by inhibiting NLRP3 inflammasome activation.

By contrast, IL-1 blockers, such as anakinra, and NLRP3 inhibitors, such as IFN1a, showed no significant clinical benefits to patients with CD [151,152].

### 6.2. Contradictory Results on the Role of NLRP3 Inflammasome Regulation in Colitis

There are several reasons that could explain the discrepancies in the results of studies on caspase-1. The first is the presence or absence of caspase-11, which is involved in the assembly of non-canonical inflammasomes [35]. The *Caspase-1* and *Caspase-11* genes are too close in the genome to be separated by recombination. As a result, previously published caspase-1 KO mouse models lacked both caspase-1 and caspase-11. Macrophages from Casp1^−/−^Casp11^129mt/129mt^ mice did not secrete IL-1β after stimulation with ATP, *C. difficile* toxin B, cholera toxin B, and *E. coli*, suggesting an important role for caspase-1 in IL-1β production. In contrast, caspase-11 is required for macrophage cell death mediated by non-canonical inflammasomes. The second cause is the involvement of the microbiota. Błażejewski et al. [153] generated caspase-1 KO and caspase-11 KO mice and rederived them into an enhanced barrier facility by embryo transfer to standardize the microbiota. The results of this study indicated that canonical caspase-1 activation, but not that of caspase-11, was responsible for the exacerbation of DSS-induced colitis.

In addition, the results on DSS colitis emphasize the importance of normalizing the microbiota between genotypes to reveal the physiological effect of a gene on DSS colitis [154,155]. The dysbiotic microbiota observed in NLRP6 KO or ASC KO mice enhanced colitis development in WT mice. WT mice co-housed with these inflammasome KO mice developed a more severe DSS-induced colitis than single-housed WT, and this effect was attributed to the horizontal transfer of colitogenic microbiota derived from the Nlrp6- or ASC-deficient mice to WT mice [116]. The use of non-littermates may lead to misleading results due to microbiota differences that are not related to host genetics; therefore, using littermate controls would be a more accurate approach to prove the involvement of the host genetic factor. Accordingly, a study performed using a littermate-control approach showed that, contrary to previous reports, NLRP6 and ASC were not involved in shaping the microbiota composition [154]. The studies on the relationship between NLRP3 inflammasome and colitis used different methods to control possible differences in the microbiota composition, such as cohousing [83,144], littermate [93,115], and embryo transfer [153]; however, some studies did not utilize these controls. Therefore, a thorough interpretation of the results is needed. The relationship between the effects of NLRP3 inflammasome-related KO mice on colitis and the methods to control the microbiota composition are shown in Table 1.

## 7. Conclusions

Many studies have shown that the NLRP3 inflammasome plays an important role in colitis, but the interpretations of the results are still controversial. The controversies may be due to various factors, such as differences in genetic backgrounds of mice and humans, compositions of microbiotas, selected models of colitis, and regiospecific features of the NLRP3 inflammasome. An appropriate level of NLRP3 inflammasome activation is believed to be required for the maintenance of intestinal homeostasis, and thus, further research is required to explore the suitable application of NLRP3 inflammasome inhibitors in the treatment of IBD.

## Figures and Tables

**Table 1:** Relationship between the effect of NLRP3 inflammasome-related KO mice on colitis and the method used to control the microbiota composition.

Mouse Model	Method to Control Microbiota Composition	Trigger	Effect on Colitis Compared to WT	References
NLRP3 KO	Littermate, Cohousing	DSS	Exacerbated	[115]
NLRP3 KO	Littermate, Cohousing	TNBS	Exacerbated	[115]
NLRP3 KO	Cohousing	oxazolone	Exacerbated	[83]
NLRP3 KO	Cohousing	DSS	Ameliorated	[144]
NLRP3 KO	Cohousing	TNBS	Ameliorated	[144]
NLRP3 KO	None	DSS	Exacerbated	[94]
NLRP3 KO	None	DSS	Ameliorated	[80]
NLRP3 KO	None	titanium dioxide nanoparticles	Ameliorated	[145]
ASC KO	None	DSS	Exacerbated	[94]
ASC KO	None	DSS	Exacerbated	[116]
ASC KO	None	C. difficile infection	Exacerbated	[81]
Caspase-1 KO	Embryo transfer, Cohousing	DSS	Ameliorated	[153]
Caspase-1 KO	Cohousing	oxazolone	Exacerbated	[83]
Caspase-1 KO	None	DSS	Exacerbated	[94]
Caspase-1 KO	None	DSS	Exacerbated	[141]
Caspase-1 KO	None	DSS	Ameliorated	[138]
IL-18 KO in intestinal epithelial cells	Littermate, Cohousing	DSS	Ameliorated	[93]
IL-18r1 KO in intestinal epithelial cells	Littermate, Cohousing	DSS	Ameliorated	[93]
Gasdermin D KO	Littermate, Cohousing	DSS	Exacerbated	[100]
Gasdermin D KO	Littermate	DSS	Ameliorated (compared to heterozygous control littermates gasdermin D^+/−^)	[99]

NLRP3, NOD-like receptor family pyrin domain-containing 3; ASC, apoptosis-associated speck-like protein containing a caspase recruitment domain; IL, interleukin; DSS, dextran sulfate sodium; TNBS, 2,4,6-trinitrobenzene sulfonic acid; WT, Wild type.

## Abbreviations

NLRP3, 1, 6, 12	NOD-like receptor family pyrin domain-containing 3, 1, 6, 12
PRR	Pattern recognition receptor
PAMP	Pathogen-associated molecular pattern
DAMP	Danger-associated molecular pattern
CAPS	Cryopyrin-associated periodic syndrome
CARD	Caspase recruitment domain
ASC	Apoptosis-associated speck-like protein containing a caspase recruitment domain
IL	Interleukin
NLR	NOD-like receptor
PYD	Pyrin domain
IBD	Inflammatory bowel disease
KO	Knockout
TLR	Toll-like receptor
RLR	Retinoic acid-inducible gene-I-like receptor
AIM2	Absent in melanoma 2
ALR	Absent in melanoma 2-like receptor
PYHIN	Pyrin and HIN domain
NLRC4	NOD-like receptor family caspase recruitment domain-containing 4
NACHT	Neuronal apoptosis inhibitory protein, MHC class II transcription activator, incompatibility locus protein from *Podospora* anserina, and telomerase-associated protein
NAIP	Neuronal apoptosis inhibitory protein
CIITA	MHC class II transcription activator
HET-E	Incompatibility locus protein from *Podospora* anserina
TP1	Telomerase-associated protein
LRR	Leucine-rich repeat
NAIP	Neuronal apoptosis inhibitory protein
IL-1R	Interleukin-1 receptor
NF-κB	Nuclear factor-kappa B
T3SS/T4SS	Type III/IV secretion system
MDP	Muramyl dipeptide
ROS	Reactive oxygen species
BTK	Bruton’s tyrosine kinase
JNK	c-Jun N-terminal kinase
NEK7	NIMA-related kinase 7
TAK1	TGF-β-activated kinase 1
GBP5	Guanylate-binding protein 5
CaSR	Calcium-sensing receptor
CD	Crohn’s disease
LPS	Lipopolysaccharide
FMF	Familial Mediterranean fever
UC	Ulcerative colitis
SNP	Single-nucleotide polymorphism
TNF	Tumor necrosis factor
Th	T helper
DSS	Dextran sulfate sodium
IFN	Interferon
IL-18BP	Interleukin-18-binding protein
NK	Natural killer
IEC	Intestinal epithelial cell
ATG	Autophagy-related gene
Treg	T regulatory
SCFA	Short-chain fatty acid
PBMC	Peripheral blood mononuclear cell
WT	Wild type
AOM	Azoxymethane
STAT	Signal transducer and activator of transcription
TNBS	2,4,6-trinitrobenzene sulfonic acid
